# The Sequential Radiographic Effects of Preoperative Chemotherapy and (Chemo)Radiation on Tumor Anatomy in Patients with Localized Pancreatic Cancer

**DOI:** 10.1245/s10434-020-08427-4

**Published:** 2020-04-07

**Authors:** Giampaolo Perri, Laura Prakash, Giuseppe Malleo, Andrea Caravati, Gauri R. Varadhachary, David Fogelman, Shubham Pant, Eugene J. Koay, Joseph Herman, Laura Maggino, Michele Milella, Michael Kim, Naruhiko Ikoma, Ching-Wei Tzeng, Roberto Salvia, Jeffrey E. Lee, Claudio Bassi, Matthew H. G. Katz

**Affiliations:** 1 Department of Surgical Oncology, Unit 1484, The University of Texas MD Anderson Cancer Center, Houston, TX USA; 2 Department of General and Pancreatic Surgery, Verona University Hospital, Verona, Italy; 3 Department of Gastrointestinal Medical Oncology, The University of Texas MD Anderson Cancer Center, Houston, TX USA; 4 Department of Radiation Oncology, The University of Texas MD Anderson Cancer Center, Houston, TX USA; 5 Department of Gastrointestinal Medical Oncology, Verona University Hospital, Verona, Italy

## Abstract

**Background:**

The incidence and magnitude of indicators of radiographic response of pancreatic cancer to systemic chemotherapy and (chemo)radiation administered prior to anticipated pancreatectomy are unclear.

**Methods:**

Sequential computed tomography scans of 226 patients with localized pancreatic cancer who received chemotherapy consisting of 5-fluorouracil, leucovorin, oxaliplatin, and irinotecan (FOLFIRINOX) or gemcitabine and nanoparticle albumin-bound paclitaxel (GA) with or without (chemo)radiation and who subsequently underwent surgery with curative intent from January 2010 to December 2018 at The University of Texas MD Anderson Cancer Center and Verona University Hospital were re-reviewed and compared.

**Results:**

Overall, 141 patients (62%) received FOLFIRINOX, 70 (31%) received GA, and 15 (7%) received both; 164 patients (73%) received preoperative (chemo)radiation following chemotherapy and prior to surgery; and 151 (67%), 70 (31%), and 5 (2%) patients had Response Evaluation Criteria in Solid Tumors (RECIST) stable disease, partial response, and progressive disease, respectively. The tumors of 29% of patients with borderline resectable or locally advanced cancer were downstaged after preoperative therapy. Radiographic downstaging was more common with chemotherapy than with (chemo)radiation (24% vs. 6%; *p* = 0.04), and the median tumor volume loss after chemotherapy was significantly greater than that after (chemo)radiation (28% vs. 17%; *p* < 0.01).

**Conclusions:**

Less than one-third of patients treated with FOLFIRINOX or GA with or without (chemo)radiation experienced either RECIST partial response or radiographic downstaging prior to surgery. The incidence of tumor downstaging was higher and the magnitude of tumor volume loss was greater following chemotherapy than after (chemo)radiation.

More than 30% of all patients who present with pancreatic ductal adenocarcinoma (PDAC) do so with infiltrative, borderline resectable (BR), or locally advanced (LA) tumors without distant metastases. Margin-negative (R0) pancreatectomy, the treatment modality most likely to lead to long-term local control and survival in patients with PDAC, is unlikely for most of such patients.^1^ Partly in an attempt to reduce the size or anatomic extent of primary tumors, and thereby improve the ability of surgeons to achieve R0 resection, patients with large and/or invasive pancreatic tumors have increasingly undergone sequential chemotherapy and/or (chemo)radiation prior to pancreatectomy. This represents the current standard of care for the treatment of BR PDAC.^2^,^3^ Whereas the role of surgery following chemotherapy and/or (chemo)radiation is limited in patients who present with LA cancers, such patients may also be considered for resection in the event of significant tumor downstaging.

Historically, anatomic downstaging was distinctly rare following the administration of gemcitabine, an agent associated with a radiographic response rate lower than 10% in patients with metastatic disease.^4^ Indeed, we previously identified only one patient with BR PDAC whose disease was downstaged to radiographically resectable among 122 patients administered sequential gemcitabine-based chemotherapy and (chemo)radiation prior to anticipated surgical resection.^5^ However, the chemotherapy regimens now routinely delivered to patients with advanced PDAC, i.e. 5-fluorouracil, leucovorin, oxaliplatin, and irinotecan (FOLFIRINOX) and gemcitabine plus nanoparticle albumin-bound paclitaxel (GA), are associated with radiographic response rates of 32% and 23%, respectively, in the metastatic setting.^4^,^6^ Whether these relatively favorable response rates translate into meaningful changes in the tumor anatomy in patients hoping to undergo subsequent resection of localized PDAC is unclear. Furthermore, whereas we have shown that preoperative radiation therapy may improve rates of R0 resection and local control over chemotherapy alone, whether subsequent (chemo)radiation further downstages tumors previously treated with these systemic regimens has yet to be established.^7^,^8^

The primary aim of this study was to characterize the changes in primary pancreatic tumor size and/or anatomic extent that occur in response to systemic treatment with FOLFIRINOX and GA as well as subsequent (chemo)radiation. To that end, we evaluated the computed tomography (CT) scans of PDAC patients who underwent surgery with curative intent following therapy at two institutions.

## Methods

The Institutional Review Boards of The University of Texas MD Anderson Cancer Center (IRB #PA18-1093) and Verona University Hospital (PAD-R, n. 1101cesc) approved this retrospective study. Individual informed consent was waived. The two centers’ prospectively maintained pancreatic tumor databases were used to identify consecutive patients who (1) received at least three cycles of preoperative chemotherapy with FOLFIRINOX and/or GA as their first line of therapy; (2) underwent surgery with curative intent for localized PDAC from January 2010 to December 2018; and (3) had both pretreatment and preoperative CT scans available for review. Among 240 patients who met these criteria, 14 patients were subsequently excluded from analysis: 5 patients who had a final diagnosis of PDAC arising in an intraductal papillary mucinous neoplasm, 4 who had a baseline CT scan showing severe acute pancreatitis or no visible mass, and 5 in whom surgical resection was aborted for a reason other than oncologic (e.g. retroperitoneal fibrosis, liver cirrhosis).

### Preoperative Therapy and Surgery

Prior to initiation and following the completion of preoperative chemotherapy and (chemo)radiation, anatomic disease staging for all patients was accomplished using multidetector CT or magnetic resonance imaging (MRI) and standard protocols optimized for imaging pancreatic tumors. Multiplanar reconstructions were used as necessary to visualize the vascular anatomy of each tumor.

All treatment decisions were made by the multidisciplinary teams at both centers. Systemic chemotherapy was routinely recommended as primary therapy to patients with a BR or LA tumor, and was also generally recommended to all patients with a resectable tumor at MD Anderson Cancer Center. At Verona University Hospital, it was administered more selectively to such patients, primarily to those with a radiographic interface between their tumor and superior mesenteric vein or portal vein. However, at both institutions, systemic chemotherapy was routinely administered to patients with a resectable tumor and one or more of the following: (1) imaging studies demonstrating findings suspicious but not diagnostic for extrapancreatic disease; (2) a depressed performance status or significant comorbidity profile; and (3) a carbohydrate antigen (CA) 19-9 level (in the absence of jaundice) suggestive of disseminated cancer.

Systemic chemotherapy consisting of FOLFIRINOX and/or GA was administered to all evaluated patients. (Chemo)radiation therapy was administered selectively at both centers and was delivered more frequently to patients with tumors who had any degree of mesenteric vascular involvement. Radiation therapy consisted of external-beam radiation therapy (total dose, 50.4 Gy delivered over 6 weeks, or 30 Gy delivered over 2 weeks) with concurrent administration of 5-fluorouracil, capecitabine, or gemcitabine or stereotactic body radiation therapy (SBRT) delivered over 5 days without a radiosensitizer. Within 8 weeks after completing preoperative therapy, the patients’ disease was clinically and radiographically restaged. Patients without evidence of disease progression and with adequate performance statuses were considered for surgical resection. Pancreatoduodenectomy, distal pancreatectomy, or total pancreatectomy was performed using standardized techniques at both centers.^9^,^10^

### Histopathologic Analysis

Gastrointestinal pathologists used standardized protocols to evaluate all surgical specimens.^11^ R1 margin status was defined as evidence of cancer cells at the inked bile duct or pancreatic parenchymal margin or within 1 mm of the superior mesenteric artery margin.

### Radiographic Review

CT images of all patients were reviewed for this study by two research associates (GP and AC) blinded to treatments and outcomes.

**Fig. 1:**
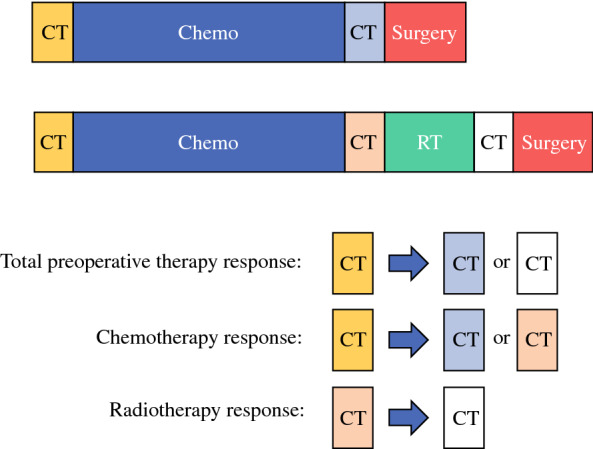
Radiographic review of CT images of pancreatic ductal adenocarcinoma patients following preoperative chemotherapy and (chemo)radiation. *Chemo* chemotherapy, *CT* computed tomography, *RT* radiation therapy

To evaluate the cumulative response to preoperative therapy, the images of each patient obtained before preoperative therapy and before surgery were compared. In addition, the radiographic changes associated with chemotherapy and (chemo)radiation were assessed independently. To evaluate the changes associated with chemotherapy alone, the pretreatment images were compared with the post-chemotherapy images (obtained prior to surgery or prior to radiation therapy). To evaluate the changes associated with radiation therapy alone, the post-chemotherapy images were compared with the preoperative images (Fig. 1).

Each tumor was staged radiographically as potentially resectable, BR, or LA according to two different classification systems—the MD Anderson Classification and the National Comprehensive Cancer Network (NCCN) guidelines.^2^,^12^ Downstaging was defined as a change from BR to resectable disease, or from LA to BR or resectable disease. Tumor size was measured using the longest (*L*) and shortest (*W*) axial diameters and the craniocaudal diameter (*H*), and the volume of each tumor was calculated using the formula for a typical ellipsoid: volume = *π*/6 × *L* × *W* × *H*.^13^ The radiographic interface between the tumor and each mesenteric vascular structure was characterized as either no contact, abutment (≤ 180° of the circumference), encasement (> 180° of the circumference), or occlusion.^14^ To measure the average attenuation in Hounsfield units, a circular region of interest encompassing one-half to two-thirds of the tumor’s area was drawn in the center of the tumor on the section with the largest surface area on each portal venous phase CT images. To characterize radiographic changes associated with preoperative therapy, the volume of the primary tumor, interface between the tumor and each mesenteric vascular structure, and tumor attenuation on pretreatment images were compared with those on post-treatment images for each patient. The change in tumor volume after preoperative treatment was calculated as the percentage of the baseline volume. Changes were also described using the modified Response Evaluation Criteria in Solid Tumors (RECIST; version 1.1).^15^ Progressive disease (PD) was defined as an increase of at least 20% in the primary tumor’s largest dimension (or absolute increase ≥ 5 mm); a partial response (PR) was defined as a decrease of at least 30% in the primary tumor’s largest dimension; stable disease (SD) was defined as an increase or decrease in tumor size insufficient to qualify as PD or a PR, respectively; and complete response (CR) was defined as total disappearance of the primary tumor.

### Carbohydrate Antigen 19-9 Level

Serum CA 19-9 levels (normal range, 0–37 U/mL) were measured prior to and following treatment. Patients in whom the CA 19-9 level was lower than 1 U/mL both prior to and following treatment were defined as nonproducers.

### Statistical Analysis

Continuous data were expressed as medians and ranges, whereas categorical data were expressed as frequencies and percentages. Continuous variables were compared using a *t* test if normally distributed and a nonparametric Mann–Whitney *U* test if not. Categorical variables were compared using a Pearson Chi square test (or Fisher’s exact test when appropriate). Statistical analyses were performed using the SPSS software program version 24.0 (IBM Corporation, Armonk, NY, USA), and *p* values < 0.05 were considered significant. All *p* values were two-sided.

## Results

We analyzed a total of 226 patients who underwent surgery following at least three cycles of FOLFIRINOX or GA with or without subsequent (chemo)radiation. Clinical characteristics of the study population are listed in Table 1. Overall, 141 patients (62%) received FOLFIRINOX, 70 (31%) received GA, and 15 (7%) received both; 164 patients (73%) received preoperative (chemo)radiation following chemotherapy and prior to surgery; and (chemo)radiation was delivered to 90 patients (63%) who presented with a resectable tumor and 74 patients (90%) with either a BR or LA tumor (MD Anderson classification).

**Table 1:** Demographic, clinical, and pathologic data for all patients [*n* = 226]

Characteristic	*n* (%)
Center	
MD Anderson	183 (81)
Verona University Hospital	43 (19)
Sex	
Female	97 (43)
Male	129 (57)
Median age at diagnosis, years (range)	64 (33–85)
Median BMI (range)	26 (16–44)
Median baseline CA 19-9 level, U/mL (range)	153 (1–9000)
Preoperative therapy	
Chemotherapy + (C)RT	164 (73)
Chemotherapy only	62 (27)
Chemotherapy regimen	
FOLFIRINOX	141 (62)
FOLFIRINOX + GA	15 (7)
GA	70 (31)
Median number of chemotherapy cycles (range)	6 (3–18)
Radiation dose [*n* = 164 patients]	
30 Gy	37 (22)
50.4 Gy	103 (63)
SBRT	24 (15)
Radiographic stage (MD Anderson Classification)	
R	144 (64)
BR	49 (22)
LAPC	33 (14)
Radiographic stage (NCCN guidelines)	
R	130 (57)
BR	63 (28)
LAPC	33 (15)
Tumor site	
Head/neck	182 (81)
Body/tail	44 (19)
Surgery outcome	
Resected	193 (85)
Aborted	33 (15)
Operation performed [*n* = 193 patients]	
PD	154 (80)
DP	32 (16)
TP	7 (4)
Venous resection [*n* = 193 patients]	
Yes	106 (55)
No	87 (45)
Lymph node status [*n* = 192 patients]	
Negative	80 (42)
Positive	112 (58)
Median number of lymph nodes examined (range)	29 (7–71)
Median tumor size, cm (range)	2.8 (0–8.0)
R status [1 mm; *n* = 193 patients]	
Negative	121 (63)
Positive	49 (25)
Not reported^a^	23 (12)

^a^ No tumor at ink, but no superior mesenteric artery margin distance recorded

*BMI* body mass index, *CA* carbohydrate antigen, *(C)RT* (chemo)radiation, *SBRT* stereotactic body radiation therapy, *NCCN* National Comprehensive Cancer Network, *BR* borderline resectable, *R* resectable, *LAPC* locally advanced pancreatic cancer, *PD* pancreatoduodenectomy, *DP* distal pancreatectomy, *TP* total pancreatectomy, *FOLFIRINOX* 5-fluorouracil, leucovorin, oxaliplatin, and irinotecan, *GA* gemcitabine plus nanoparticle albumin-bound paclitaxel

Changes in radiographic characteristics that occurred in association with preoperative chemotherapy and (chemo)radiation are reported in Table 2.

**Table 2:** Radiographic indicators of response to preoperative therapy

	Overall response to preoperative therapy [*n* = 226]	Response to chemotherapy alone [*n* = 226]	Response to (chemo)radiation alone [*n* = 159]	*p* Value
Radiographic stage (MD Anderson Classification)^a^				**0.04**
Upstaged	5 (2)	3 (1)	2 (1)	
No change	197 (87)	203 (90)	153 (96)	
Downstaged	24 (29^b^)	20 (24^b^)	4 (6^b^)	
Radiographic stage (NCCN guidelines)^a^				**0.03**
Upstaged	6 (3)	5 (2)	1 (1)	
No change	194 (86)	200 (89)	153 (96)	
Downstaged	26 (27^b^)	21 (22^b^)	5 (6^b^)	
Treatment response (RECIST 1.1)				0.50
CR	0	0	0	
PR	70 (31)	46 (20)	25 (16)	
SD	151 (67)	176 (78)	131 (82)	
PD	5 (2)	4 (2)	3 (2)	
Decreased tumor volume				0.07
No	46 (20)	62 (27)	31 (19)	
Yes	180 (80)	164 (73)	128 (81)	
Median %Δvol (range)	36% (− 159% to 99%)	28% (− 177% to 97%)	17% (− 127% to 95%)	**< 0.01**

Data are expressed as *n* (%) unless otherwise indicated

Bold characters indicate statistical significance

^a^Upstaging: any change from resectable to BR or LA cancer, or from BR to LA cancer. Downstaging: any change from LA cancer to either BR or resectable cancer, or from BR to resectable cancer

^b^Percentage of patients with BR or LA pancreatic cancer

*NCCN* National Comprehensive Cancer Network, *RECIST* Response Evaluation Criteria in Solid Tumors, *CR* complete response, *PR* partial response, *SD* stable disease, *PD* progressive disease,  *%Δ*_*vol*_ change in tumor volume, *BR* borderline resectable, *LA* locally advanced

### Radiographic Stage

**Fig. 2:**
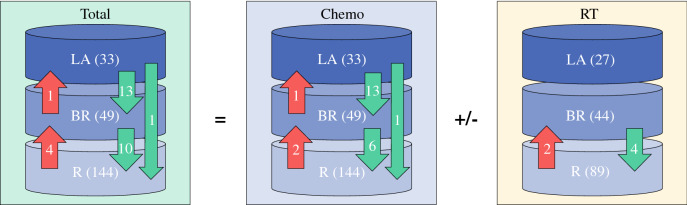
Changes in radiographic stage that occurred in association with preoperative therapy. *Chemo* chemotherapy, *RT* radiation therapy, *LA* locally advanced, *BR* borderline resectable, *R* resectable

The MD Anderson radiographic stage of 197 tumors (87%) did not change following the administration of preoperative therapy, whereas five tumors (2%) were upstaged due to local progression. The tumors of 24/82 patients (29%) who had BR or LA cancer were downstaged after preoperative therapy. We observed radiographic downstaging of BR and LA tumors after chemotherapy in 20 patients (24%) and after subsequent (chemo)radiation in 4 patients (6%) [Fig. 2]. These rates were similar when we staged the tumors using the NCCN criteria.

### Response Evaluation Criteria in Solid Tumors (RECIST) 1.1 Response

According to RECIST 1.1, 151 (67%), 70 (31%), and 5 (2%) patients had SD, PR, and PD, respectively, after preoperative therapy. We observed no significant differences in responses assessed using RECIST 1.1 after (chemo)radiation or chemotherapy (*p* = 0.5).

### Tumor Volume

The volume of 180 primary tumors (80%) decreased after preoperative therapy. Patients experienced a median baseline tumor volume loss of 36% over the entire preoperative treatment course. Loss of volume was equally common following chemotherapy and (chemo)radiation (73% vs. 81%; *p* = 0.7); however, the median tumor volume loss after chemotherapy was significantly greater than that after (chemo)radiation (28% vs. 17%; *p* < 0.01). Notably, volume loss following chemotherapy was similar between patients who received only chemotherapy and those who received chemotherapy followed by (chemo)radiation (28% vs. 28%; *p* = 0.2). In addition, there was no difference in terms of median volume loss between 50.4 Gy and 30 Gy (chemo)radiation (20% vs. 16%; *p* = 0.2), and between ‘conventional’ (chemo)radiation and SBRT (19% vs. 11%; *p* = 0.2).

### Radiographic and Serologic Characteristics of Patients with Unresected Tumors

After receiving chemotherapy with or without (chemo)radiation, 193 patients (85%) underwent resection of their primary tumors and regional lymph nodes, whereas 33 (15%) did not due to intraoperative identification of metastases (*n* = 25, 11%) or a local tumor anatomy that precluded resection (*n* = 8, 4%). Patients who did not undergo resection were similar to those who did in terms of treatment response (according to RECIST 1.1), decrease in tumor volume, increase in tumor attenuation, changes in tumor-vessel interfaces, and other potential indirect radiographic signs of tumor response to preoperative therapy (all *p* > 0.05) [Table 3]. The median post-treatment CA 19-9 level in patients whose surgery was aborted was higher than that in those in whom resection was completed (37 U/mL vs. 25 U/mL; *p* = 0.04).

**Table 3:** Potential radiographic and serologic indicators of pancreatic ductal adenocarcinoma resectability following preoperative therapy

Indicator	Total [*n* = 226]	Resected		*p* Value
		Yes [*n* = 193]	No [*n* = 33]	
*Radiographic*				
RECIST 1.1				0.20
CR	0	0	0	
PR	70 (31)	62 (89)	8 (11)	
SD	151 (67)	128 (85)	23 (15)	
PD	5 (2)	3 (60)	2 (40)	
Decreased tumor volume				0.20
No	46 (20)	37 (80)	9 (20)	
Yes	180 (80)	156 (87)	24 (13)	
Median %Δvol (range)	36% (− 159 to 99%)	39% (− 159% to 99%)	32% (− 85 to 90%)	0.30
Median increase in attenuation, HU (range)	3 (− 49 to 89)	4 (− 49 to 89)	− 4 (− 34 to 56)	0.20
SMV/PV interface^a^				0.20
Progression	10 (4)	7 (70)	3 (30)	
No change	182 (81)	154 (80)	28 (15)	
Improvement	34 (15)	32 (94)	2 (6)	
SMA/CHA/CA interface^a^				0.20
Progression	5 (2)	3 (60)	2 (40)	
No change	207 (92)	177 (85)	30 (15)	
Improvement	14 (6)	13 (93)	1 (7)	
Median GC venous trunk diameter, mm (range)	5 (2–14)	5 (2–14)	6 (4–10)	0.06
Median MPD diameter, mm (range)	5 (1–18)	5 (1–18)	4 (1–13)	0.80
*Serologic*				
Median preoperative CA 19-9 level, U/mL (range)	29 (1–3039)	25 (1–2344)	37 (1–3039)	**0.04**
Preoperative CA 19-9 level (cut-off, 37 U/dL)				0.30
Not expressed	9 (4)	8 (89)	1 (11)	
Elevated	85 (38)	69 (81)	16 (19)	
Normal	132 (58)	116 (88)	16 (12)	
CA 19-9 level response after therapy (from baseline)				0.30
Not expressed	9 (4)	8 (89)	1 (11)	
Normal to elevated	3 (1)	0	3 (100)	
Remained elevated	82 (36)	66 (80)	16 (20)	
Remained normal	46 (20)	38 (83)	8 (17)	
Elevated to normal	86 (38)	78 (91)	8 (9)	

Data are expressed as *n* (%) unless otherwise indicated

Bold characters indicate statistical significance

^a^Classification for tumor-vessel interface: no contact, abutment (≤ 180° of the circumference), encasement (> 180° of the circumference), or occlusion. Progression: any change from lower classification to higher classification. Improvement: any change from higher classification to lower classification

*RECIST* Response Evaluation Criteria in Solid Tumors, *CR* complete response, *PR* partial response, *SD* stable disease, *PD* progressive disease,  *%Δ*_*vol*_ change in tumor volume, *HU* Hounsfield units, *SMV* superior mesenteric vein, *PV* portal vein, *SMA* superior mesenteric artery, *CHA* common hepatic artery, *CA* celiac artery, *GC* gastro-colic, *MPD* main pancreatic duct, *CA* carbohydrate antigen

## Discussion

We previously demonstrated that a reduction in the size or anatomic extent of localized PDAC is uncommon following preoperative administration of gemcitabine-based multimodality therapy.^5^ In this study, we sought to examine putative markers of radiographic response in a cohort of patients who received FOLFIRINOX or GA with or without subsequent (chemo)radiation. We found that less than one-third of patients administered these regimens with or without subsequent (chemo)radiation prior to laparoscopy or laparotomy with curative intent experienced either PR, according to RECIST 1.1, or radiographic downstaging, even though 80% of their tumors decreased in volume to some degree. Furthermore, although the incidence of tumor volume loss following chemotherapy was similar to that following subsequent (chemo)radiation, the magnitude of tumor volume loss was greater and the incidence of downstaging was higher following chemotherapy than after subsequent (chemo)radiation.

In this study, we examined changes in tumor volume, anatomic stage, and treatment response after administration of preoperative therapy for PDAC. The clinical significance of such findings has been challenged in the past, primarily in studies such as our own that have clearly demonstrated that a radiographic response is not necessarily required to achieve R0 resection of a BR or LA cancer.^5^,^16^,^17^ Nonetheless, these metrics have profound clinical utility. For example, the post-treatment radiographic stage is a robust predictor of the need for vascular resection and clinically significant vascular invasion.^14^ In addition, radiographic changes appear to be clinical readouts of the efficacy of treatments delivered prior to surgery. We recently demonstrated that PR (according to RECIST 1.1) and radiographic loss of tumor volume are both strongly associated with the validated pathologic metric of pathologic major response. Furthermore, patients who experienced pathologic major responses had strikingly longer median overall survival durations than patients in whom pathologic response to preoperative therapy was less robust.^18^

In this study, we independently assessed the associations of chemotherapy and (chemo)radiation with each putative metric of treatment response. We found a greater magnitude of tumor volume loss and a higher incidence of radiographic downstaging following chemotherapy than following subsequent (chemo)radiation. Specifically, the tumors in only 6% of the patients with LA or BR cancer were further downstaged by (chemo)radiation administered after chemotherapy (Fig. 2). Although these findings suggest a limited role for radiation therapy following induction chemotherapy in this setting, they must be viewed with caution. First, radiation therapy may be associated with tissue edema that could mask the extent to which a tumor decreases in size or anatomic extent. Second, (chemo)radiation appears to have clinically relevant effects on pancreatic cancer that cannot be visualized radiographically. For example, we have shown that (chemo)radiation reduces lymph node metastasis, maximizes the distance between cancer cells and the superior mesenteric artery margin and increases local cancer control even though it may not prolong survival.^7^,^8^,^19^ Regardless, our results question the ability of radiation therapy to significantly reduce the volume or anatomic extent of a tumor following induction chemotherapy with FOLFIRINOX or GA. These findings therefore suggest that administering radiation therapy following chemotherapy for the primary purpose of further ‘shrinking’ a tumor away from the vessels and facilitate resection is misguided.

In a secondary analysis of the CT scans of these patients, we could not identify any radiographic measures that could be reliably used to indicate occult metastases in patients with otherwise localized cancer. Therefore, at present, surgery with curative intent is a reasonable approach for physiologically robust patients without radiographic evidence of disease progression during preoperative therapy. Novel biomarkers may help avoid unnecessary surgical exploration or even pancreatectomy in patients unlikely to benefit from surgery, and are clearly needed. In the meantime, proposed strategies to reduce the rate of unnecessary laparotomies due to unanticipated metastases include staging laparoscopy prior to laparotomy (at least in patients with CA 19-9 levels or radiographic findings suspect for disseminated disease) and/or routine preoperative MRI.^20^,^21^

The primary limitation of this study was that all patients underwent laparoscopy or laparotomy and thus were already selected on the basis of their radiographic response. Certainly, some patients not included had radiographically evident metastatic disease during preoperative therapy. However, the primary findings of this study, i.e. that PDAC in fewer than one-third of patients was downstaged after treatment with FOLFIRINOX or GA with or without (chemoradiation) and that radiographic measures of response were generally more robust following chemotherapy than after (chemo)radiation, would only be more pronounced if we had included patients with metastatic progression. In addition, we evaluated radiographic response to (chemo)radiation only in patients who had already received preoperative chemotherapy. The responses to (chemo)radiation delivered de novo may have been more robust; however, (chemo)radiation is typically delivered to patients with localized PDAC only after induction chemotherapy to improve patient selection for this local treatment modality.^22^,^23^ Finally, patients in this study received a median of six preoperative cycles of chemotherapy, and most received standard chemoradiation. The extent to which longer courses of chemotherapy or different radiation therapy regimens would improve radiographic measures of response over those described herein is unclear.

## Conclusions

We found that despite the use of FOLFIRINOX or GA, radiographic downstaging of PDAC occurred in less than one-third of patients administered systemic chemotherapy prior to anticipated pancreatectomy. Furthermore, the magnitude of tumor volume loss was greater and the incidence of tumor downstaging was higher following chemotherapy than following subsequent (chemo)radiation. These findings should be used to set expectations with respect to the possible effects of chemotherapy and (chemo)radiation in PDAC patients prior to pancreatectomy.
